# 

**Published:** 1909-03-15

**Authors:** 


					﻿Dental
Register
NELVILLE S. HOFF, lAiúor'. Q J
Ann Arbor, Mich.
Vol. LXIII —MARCH, 1909- No. 3
SAMUEL A. CROCKER & CO., Publishers
OHIO DENTAL-AND SURGICAL DEPOT
35 W. FIFTH STREET, CINCINNATI, 0.
FRIGE, ONE DOLLAR A YEAR
Entered at the Posloffice at Cincinnati, O, as second-class mail matter
				

